# Valedictory Address to the Graduating Class

**Published:** 1855-04

**Authors:** James Taylor


### VALEDICTORY ADDRESS TO THE GRADUATING CLASS.

BY DR. JAMES TAYLOR.

Delivered February 23d, 1855.

In the progress of events, it is well occasionally to stop and look back upon the past, to carefully note down the incidents which go to make up the history of our enterprise.

In this way we may scan with scrutiny the errors committed, and fortify ourselves with the wisdom of experience for the future.

History is a collection of facts, a "narration of events in the order in which they happened, with their causes and effects."

Dental Science has its history, and this runs back much farther than is generally supposed. Egypt, in her palmy days, had her hereditary dental practitioners—their skill lies buried with the ruins of her empire, and the crumbling rubbish which occasionally unfolds much progress in science, reveals but little to enlighten us in our speciality.

Imperial Rome, in her proudest days of pomp and power, knew the use of our art, and many of her fair and stately dames were indebted to dental skill for their attractive smiles.

But we intend not this evening to draw from the *distant past* a theme for the present occasion. True, there is much which might interest and instruct; yet it is not always best to trace back genealogy to so distant a period. We love more to view our science as nature's *nobleman*, who comes forth without any heralding of the past, and takes his position in the world by the aid of his own powerful genius—the trappings of the worn out and decrepit great lay in tatters at his feet, and he robes himself with the habiliments of a progressive age. High on the temple of Fame, he views an unsculptured tablet, and by the aid of an indomitable will and energy of purpose, he determines to engrave his name on it.

Dental Science only of late has been able to shake off the shackles which had bound her to the past, and present herself in her own attire. To rear for herself a name and *monument* of her progress. To collect more from the present than the past—mementoes of her skill. To inspire her sons with a zeal and ardor before unknown.

Let us, however, very briefly allude to those events which make up this history. In doing this, we need not go back farther than the recollection of those present. We need not shake off the dust of ages to trace out this history. We come to our own country, to our native land; and even to the vigorous Buckeye State.

In 1827, now scarce 28 years ago, and this city had but two Dental Practitioners. The whole State could not claim half a dozen.

At this period there was not a Dental Association, Periodical or College in the United States. The American Journal of Dental Science is the pioneer in this department, and was first published in 1839. Prof. C. A. Harris, of Baltimore, and Dr. E. Parmley, of New York, had the editorial management of the Journal, and discharged their duties with such ability that it soon became a popular periodical with the profession, and we believe to this day has been well sustained.

The profession, indeed, was ready to throw off the incubus of secrecy, which had been so long trammelling her progress, and needed just such a channel of inter-communication. Old Fogyism, it is true, in some instances became alarmed, and feared lest their secrets and craft would be taken from them. Yet the spirit of the age was aroused; public good, and the interest of true science demanded light, and there were stout hearts and willing hands ready to lift the drapery which hid from view, every secret of the art.

Some of us will never forget the monthly greetings of the Journal. How quick its pages were scanned for something new; and the half dozen lines of editorial were read and re-read, as the postscript of a lover's letter. Times have changed, and this old Friend has enlarged himself, and puts on more the dignity of age, but yet retains the vigor of ripening manhood. "May his shadow never grow less."

We are unable to give the exact time when the Dental Association of the City of New York and its auxilary of Western New York were organized. But it must have been about or before the establishment of the Journal.

On the 18th day of August, 1840, the American Society of Dental Surgeons was organized, at the American Hotel in the City of New York. Many of the most prominent of the profession in the United States, assisted in this organization, and were thus brought into free communion with each other, and who can doubt the good which has resulted therefrom.

These Associations afford at all times (and particularly at the time to which we refer) an opportunity of comparing modes of practice, and of discussing subjects which relate to Dental Science.

Such inter-communion of thought tends, very much to soften down the asperities of character, and make us view *even* each others faults with less severity. What better place to suggest an improvement, discuss its practicability, and decide on its merits.

They lead to development, urge on to experiment and a laudable ambition to excel.

During the fall of this year, 1840, the Baltimore College of Dental Surgery appears to have been opened for the reception of students. What a rapid developing of events? How suddenly Dentistry shakes off the lethargy of past ages, and with what vigor she battles for a character and name.

We can not however detail the events which crowd upon us from the East. Yet how strange that the leaven which should somewhat set in motion these elements, should have been transported from the West. Doctor Harris, one of the originators of the Baltimore College of Dental Surgery, commenced his professional career in this State. He practiced first in my native county, if not native town. He was also one of the first editors of the Journal, and assisted in the organization of the American Society. The profession owe much to his industry, energy and talent.

But we come more particularly to speak of the West. We remarked, that in 1827, there were but two Dentists in this city, and in the State, not over half a dozen. This we believe is true if we were to call every *pretender* a Dental Surgeon. There was, it is true, an occasional itinerant passing through the State. Soon, however, the number largely increased, and yet how few have ever attained any reputation as operators.

The cause of this is forcibly plain; a very large majority then entering the profession were mere mechanics, and never looked upon the profession in any other light than that of a trade. This, although not always presenting a modicum of success in a pecuniary sense; yet it has almost invariably diminished this and many such who then promised well, being evidently fitted by nature for the work, yet not having a proper appreciation of the profession they had assumed, ultimately sank down into positive insignificance, or have entirely abandoned the profession.

It would not now be difficult to trace out the history of many such, and name thus one after another the *rocks* over which they stumbled. First, an imaginary mechanical genius; this, with such, was sufficient to meet all emergencies of practice, to subserve all demands of true medical knowledge; and of what did this often consist? Simply a knowledge of *burnishing gold.*

Second, an indifference to, or repugnance to study, partial success often inflates the ignorant, they can not be made to see the importance of knowledge; they grasp at every thing by a kind of intuition; they really lay hold of nothing, but are deceived themselves, and then aim to deceive the public; they want the patience and energy of purpose to master anything. After years of supineness they find that they are foolishly ignorant.

Third, they have entered the profession ignorant of those laws and principles on which Dental Science is based, and have not the stability of purpose to master them.

Fourth, they think they are the reflection of Dental Science itself, and refuse to let the light of *that Science* shine upon them. They have really only received a few glimmering rays of light from the past, and will not believe that any thing *new* can be discovered "under the sun." They are like unpolished mirrors, which fail to reflect, or like a camera obscura without its double convex glass, the light falls into a darkened chamber.

Fifth, they believe that the perfection of Mechanical Dentistry is a fine polish, and that Medical Dentistry is bound up in two or three specific nostrums. With such, *I-S-M* always spells Orthodoxy, and although sticklers for the venerable past, they are sure to take a jaunt on every new *Ism* introduced.

It would appear that every pit fall around the temple of Dental Science had been filled by such *who* had attempted to enter its portals; although admirable riders, they are sure to be thrown as they attempt to ascend the rugged path which leads to its summit.

But we must not forget, that if the profession has changed since 1827, so has also the West. Truly, it would seem that the Magician's wand had been stretched over this great valley. The wilderness has melted away, and cities, towns and villages have risen as if by enchantment. The dense forest has given place to immense fields of grain, and the wigwam of the savage to the elegant dwellings of an indomitable and energetic race. The march of this change is still westward, and *we* appear to be just collecting and arranging the evidences of that swelling tide which sweeps on to the Pacific.

All those avocations of life, which at first were thrown *helter skelter*, by the leveling force of this mighty flood of

Western immigration, are now being arranged in order, each appropriating to itself that which it attracts by its own magnetic power. Much it is true may thus be drawn, like the dirt which clings to the gold, to where it does not properly belong. Time may wear this away, if not, that fire which purifies the gold will consume the *dross*.

If we take the period to which we have alluded, 1827, as the starting point, we will find that but little was done except by isolated individual effort in the West, for the promotion of Dental Science, for about fifteen years. The profession was itself isolated. Most of the larger towns were not permanently occupied by Dentists; some places were so rarely visited that the Dentist was scarcely classified by the people; some thinking him a real Doctor, while others could not believe him other than a Tinker. Unfortunately, the qualifications were often such as to justify this opinion. This very condition of things rendered the itinerating practice of the high minded and worthy at the least, undesirable and unpleasant. Hence the larger towns soon began to be permanently occupied, and this permanence soon gained the confidence of the public, and patronage was sure to follow. Hundreds of places once thought too small to support a Dental Practitioner, were found to be amply sufficient; and now the wealth and intelligence of the agricultural population is in many localities as reliable as the best of town practice.

This City, and others in the West, in their rapid growth opened up fine situations for the best of operators, and the wealth of our Cities, which then sought such operations in the East, now almost entirely seek it at home. A trip East, is no now considered *essential* to secure the latest impovements in Dental Science. The commingling of our profession at home, and the circulation of our Dental Periodicals, render this very pleasant recreation no longer absolutely necessary.

The changes to which we have alluded, had been such up to 1841, that even in this City the profession numbered some twenty individuals, and in the winter of that year we organized an Association for our own improvement.

This Society wishing to enlist more generally the profession, issued, in 1844, a call for a general convention. This met in August, and organized the Mississippi Valley Association of Dental Surgeons. Into this was merged as it were the Cincinnati Society.

The Mississippi Valley Association still exists in a flourishing state, and commenced its tenth annual meeting this day. It has been the means we believe of uniting in fraternal bonds, many who would otherwise have been utter strangers. It has made known to some extent the wants of the profession, and as far as appeared possible, instituted means to meet those wants. It has labored for the promotion of Dental Science, and has done much to advance friendly intercourse and courtesy among its members and the profesions generally.

This Society started, and in part sustained, for three or four years, the Dental Register of the West. Appointing a Committee yearly to edit the paper, and the expenses not met by the subscribers were liquidated by the Society. The Association thus afforded very efficient aid in the establishment of a periodical for the dessemination of knowledge.

The paper has increased in size from a fifty to an eighty page quarterly, and is still published at the same price as at first. The eighth volume is now half issued.

If the work was double the size it now is, it would be far easier to get suitable matter for its pages than at first. We name this to show the progressive spirit which is manifest in the profession. There are more who write, and a careful examination would, we think, show how much our Dental Colleges have had to do in the matter. This is at least a portion of history which will never discredit our Institutions.

In the winter of 1844–5, the Ohio College of Dental Surgery was chartered by the Legislature of this State, and went into operation the winter of 45–6, with the following individuals as a faculty or board of teachers: Drs. Cook, Rogers and Taylor.

This organization lasted two years, and fully confirmed the faculty in the views expressed in their announcement. Their feelings were expressed in the following language: "That in issuing this, their first announcement, they do it with feelings of deep responsibility." The establishment of a new institution for the diffusion of scientific knowledge, even under the most favorable auspices, is an undertaking of no small magnitude."

It was not expected that an institution of the kind should at once command favorable notice and attention. It had the prejudices of some and indifference of others to overcome. It had to contend with the conceived notion of many, that the amount of knowledge necessary to be obtained for a dentist, was so small, that a very smart *chap* could master it in a few weeks. With such, two years study and two courses of lectures was time wasted. Unfortunately this error had to be combatted not only with the student, but with many of the older practitioners, who had not yet learned that a "little knowledge is a dangerous thing."

Having no special guide to direct them in the course of instruction, the faculty had to arrange that course which the exigencies of the case seemed absolutely to require, and gradually advance the standard of dental education as the views of the profession and the interest manifested, would point out.

That we had plenty of discouragements, none will pretend to deny. Yet, with pride we would say, that the board of trustees were ever ready to sustain the institution, and gave us one with whom to counsel and advise who has never faltered in his purpose, but ever with his eye on the future, has urged us onward.

It is well, perhaps, that we cannot see in prospective all the difficulties which surround new enterprises; for if we did, many would never be undertaken. Duty impels us to engage in them, and duty should still urge us forward. But as difficulties thicken and discouragements arise, pride and ambition may rally to our support; but duty should still govern these. It is only by perseverence we may expect to gain experience, and in this way learn to master difficulties. Constancy of purpose and an unwearied zeal, will overcome many obstacles and gain ultimate success.

The chair of anatomy and physiology has been filled as follows: The first two sessions by Dr. J. W. Cook, the third session by Dr. Potter, the fourth and fifth sessions by Dr. Shotwell, and the last five sessions by Prof. T. Wood, the present incumbent.

The chair of pathology and therapeutics has been filled: The first three sessions by Dr. M. Rogers; the next four by Prof. G. Mendenhall, and the last two by the present incumbent, Prof. J. B. Smith.

The chairs more especially relating to dental practice, have been changed to meet the wants of the institution and profession.

The first session a chair of practical dentistry was considered all sufficient, and to secure more fully a thorough course in this department, Dental Pharmacy was attached. To this double post we were assigned, performing also the duties of demonstrator of Mechanical Dentistry.

The second session Dr. Slack delivered a course of lectures on Chemistry, which relieved somewhat the duties of our Chair. The lectures on Chemistry have since been more or less sustained every session by Drs. Slack, Watt, and the present session, Dr. Kellogg. During the fourth session Dr. W M. Hunter performed the duties of Mechanical Demonstrator. The fifth session, Dr. A. M. Leslie took charge of this department; and in 1850 a chair of operative Dentistry was created and filled by the appointment of Dr. A. M. Leslie, and Dr. Van Emon became demonstrator of Mechanical Dentistry. During the winter of 50 and 51, Dr. Leslie resigned, and the following spring Dr. John Allen was appointed to fill his place.

In 1852 the Chair of Mechanical Dentistry was created, and was filled by the appointment of Dr. H. R. Smith, the present incumbent. In February, 1854, Dr. J. Allen resigned, and his Chair was filled by the selection of Dr. J. Taft, the present incumbent.

We have hastily sketched the history of these changes, to show first, the somewhat instability of human affairs, and second, the expansive tendency of that which is truly practical in this Institution. This has been done to fully meet the wants of the student, and yet the other departments have not by any means been neglected. In these, the course of study has been extended from year to year, until at present in these departments a full and thorough course is given.

In relation to the Infirmary belonging to the school, we would remark, that it is capable we believe of almost indefinite extension, and can be made to meet every want of the Institution.

While the Faculty feel the necessity for opportunities of practice, yet they can not but feel that the principles on which correct practice is founded should be thoroughly understood, and that there is an absolute necessity of so dividing the time appropriated to the study of those principles and their application in practice, that mere mechanical operators will not be the result.

The Institution is specially designed to avoid or correct this difficulty, and must ever regard a proper foundation as of the utmost importance to professional life. This *under-pinning* is very unsafe and uncertain. The foundation should be broad deep and firm, sufficient to bear the additions of a long life, so that the storms of life, the new-fangled notions of the day, or the specious Isms of the times shall not be able to move the superstructure erected theron.

We would in this connection draw the attention of the profession to what we regard as an error in Dental education. In a large majority of cases, students are led to believe that the practical detail of the profession is *the* important thing to be acquired. This is all right and proper in its place and should not be neglected; but the study of the human system, and especially those organs involved in Dental disease, and the principles of Pathology are, and should be first and foremost.

A Student of Medicine would hardly think of commencing practice as soon as he commences the study. He first learns the cause and nature of disease that he may understandingly apply a remedy. The experience of the past, and common sense teach us this is right, and that in Dental Science the same is equally true.

This is a radical evil, and is almost as observable in those who have been engaged in practice as those just commencing the study, and if any thing has truly gratified us in our connection with this Institution, it is the change of views which almost universally takes place in this respect, with those who have passed through this Institution.

At first, their great aim was mechanical knowledge—at the close, they were eager for that knowledge which should govern us in practice.

One of the leading objects of this College, is to supply an educated profession, and just so far as this object is attained shall we rejoice in its prosperity. We feel that we should be doing the profession we this evening represent injustice, did we not allude to the success which has crowned their efforts in the erection of this building. A stronger proff could not well be given of the estimation in which the profession regard dental education, than the self-sacrifice which has been displayed in securing such a building devoted to this exclusive purpose. The building as it now stands, including the ground, has cost ten thousand dollars; six thousand dollars of this has already been paid in (or nearly so) by the subscription of stock, and some interest which has accrued on the stock paid in, which has liberally been appropriated to raise a sinking fund to liquidate all claims against the Institution for building purposes. Each share of stock is one hundred dollars, and bears interest at the rate of 6 per cent. per annum. This is paid by the Faculty as a rent on the building; they also paying the taxes and insurance. There is now, therefore, over fifty shares of stock subscribed and paid for. These stockholders are scattered from Pittsburgh to New Orleans, throughout this great valley, and we here take pleasure in saying that an establishment in Philadelphia, connected with the profession, very liberally took two shares.

The first purchase was the lot on which this house stands, with a temporary building erected for the purpose, and some permanent fixtures; for which thirty-eight hundred dollars was paid. Some three years since, it was found that the Institution required a larger building and more convenient rooms, and arrangements were then made to secure funds for this purpose, and at our last commencement, just one year since, a committee was appointed to have erected during the past summer this building. This Committee have succeeded as we hope, in getting just such a building as the Institution needs, and as we think sufficient to meet the wants of the profession for years to come.

We can not but regard the completion of the Ohio College of Dental Surgery, and the permanent footing thus secured for the Institution, as worthy of special notice. Its future management is in the hands of a Board of Trustees, appointed by the Legislature of this State, the stockholders and the graduates who may become members of the Ohio Dental College Association. Its fate is therefore in the hands of the profession. They are its Guardians, and that they will see to its interests is well evinced by the noble manner with which they have rallied to its support.

The profession, in this great valley of the West, may feel proud that they have led the way in the erection of a Temple, around which they may rally for the promotion of Dental Science. So far as known, it is the first building of the kind ever erected. Others have instituted Colleges for the purposes of dental education; but they have not made for themselves a *home*, where they may yearly congregate, and feel that every contribution made shall be locked up and preserved for future ages.

The Faculty feeling, as we trust, the obligations thus imposed upon us, have endeavored to adopt such rules and regulations as shall effectually secure the best possible qualifications in those who may receive the honors of the Institution.

No half way course of study is allowed, actual attendance upon the lectures is required, and critical examinations are made throughout the entire course. The tickets all must be taken, so that every matriculation is bona-fide evidence of regular studentship. Our candidates for graduation, having thus fulfilled requirements, are enabled to come forward and demand as a matter of right and merit, the highest credentials the Institution can give. We regard it the duty of the Trustees, the Faculty, and the Stockholders, to guard well the honors of the College, and make them what they purport to be; evidences of merit. Not simply an unmeaning piece of parchment, but that which shall speak to the world of true excellence, and be a pass-port wherever true Medical and Dental Science is known.

This is our object, this our aim, Dental Science demands it at our hands; for this we labor, and shall battle on and ever until ignorance and superstition is vanquished and the Diplomas of this Institution shall be known and respected, as was once the proud title of Roman Citizen. We feel that although *we* may not live to see this ourselves, yet there is *that* in the profession we now represent, which will urge on to this consummation.

This is the ninth session of the school, and our books exhibit over one hundred and thirty matriculants, and about fifty regular graduates. Many causes have operated to diminish the number of the present class; among which we may name the scarcity of money, and the fact that we have adopted the cash system, or its equivalent. Under these circumstances, we feel that the Institution is now in a more prosperous condition than ever before. It must be recollected that the number of students does not always denote the true condition of a school. We are sorry to say that the matriculating list is not always a *true* index to the real size of a class.

We have thus given a brief history of the profession in the West, embracing a short sketch of the Ohio College of Dental Surgery.

Our remarks have hence been addressed to the profession and the public.

These things Gentlemen interest you as members of an important profession. You are enabled to see the efforts which have of late been made to advance the standard of Dental attainment.

There is no department of the healing art, requiring more patient perseverance and untiring industry, than that you have chosen. Every detail of your profession requires exactness and fidelity. Your entire intercourse with those who seek your aid, should be courteous, kind, and marked with the strictest integrity. The honor and the dignity of the profession should ever be sought in all your operations; ever remembering that the profession's character is in your hands, and that the more illustrious this is made, the more useful you may become.

You have chosen your profession for life, take care that your affections are not divided. If the profession is worthy of your devotion at all, it should receive all your energies; without this you may not expect success.

Your profession is an exacting mistress, refusing her choicest favors, unless assiduously *wooed.* Yet, if this homage is laid at her feet, she will lavish her richest treasures upon you, and reward you threefold for your attention.

May she ever find you assiduous wooers, ardent lovers, and devoted defenders of her rights.

Gentlemen Graduates: — You have now received your *sealed* credentials. Its device is an open book with this motto, " Science combats disease." You now go forth to test your weapons in this great combat; may they ever be directed by those great principles on which our science is based. Thus directed and skillfully used, success will crown your efforts.

You have already been addressed in relation to those great moral principles which should be our guide through life, and above all, to those sacred truths which must test our conduct here, and by which we must be judged at that final end to which we are all tending.

These are first and paramount, and form the only sure foundation to rest upon. Be *sure* you so receive and treasure them, that however fortune may smile upon you, or the ills of life may perchance thicken upon you, they shall be your guide; they will alone form a shield around you which can protect in the temptation of prosperity or adversity of affliction.

You have been pointed to professional excellence, to the trials and aims of life, and that zeal with which you should sustain the dignity and interests of your profession. This hall, with its associations cluster around you. The past is a dream. The present, the future, what are they? We cling to the present, the chain that binds us can scarce be severed; we are rivited to this place, we linger here; time waits not for us, and the busy rattling world moves onward. The spell must be broken, for the future is in prospective, and gilded hopes, like airy castles, are beckoning you away. Sweet home, father, *mother*, sisters, brothers, wife and *loved* ones, and perhaps other *ties*, scarce revealed, send a thrill of joy and hope to your hearts. Blest Elysian, may its spell lead to happiness.

The future, then, what it is? Will it be a realization of these fond hopes, a blending of sunshine and shadow, a realization of bright anticipations.

Whether this will be so or not, you have, in part, the shaping of your future.

Duties you have to perform. These duties should be faithfully executed. Leaving the result to Him who overrules all events, to His glory, and for the good of those who trust in Him.

You leave us with the best wishes of the Faculty for your long continued usefullness and prosperity. There is not a step in your future life but will be watched with interest by your friends, as well as by the friends of science.

